# Hydrogen peroxide (H_2_O_2_) induces leukemic but not normal hematopoietic cell death in a dose-dependent manner

**DOI:** 10.1186/1475-2867-13-123

**Published:** 2013-12-23

**Authors:** Amanda Nogueira-Pedro, Thalyta Aparecida Munhoz Cesário, Carolina Carvalho Dias, Clarice Silvia Taemi Origassa, Lilian Piñero Marcolin Eça, Edgar Julian Paredes-Gamero, Alice Teixeira Ferreira

**Affiliations:** 1 Department of Biophysics, of Universidade Federal de São Paulo, R. Botucatu, 862 – 2° andar, 04062-023, São Paulo, Brazil; 2 Department of Biochemistry, Universidade Federal de São Paulo, R. Pedro de Toledo, 669 – 9° andar, 04062-023, São Paulo, Brazil; 3 Department of Medicine, Universidade Federal de São Paulo, R. Pedro de Toledo, 669 – 10° andar, 04062-023, São Paulo, Brazil; 4 IPCTRON Stem Cell Research Institute, R. Dr. Diogo de Faria, 159, 04037-000, São Paulo, Brazil; 5 Universidade Federal de São Paulo, Rua Pedro de Toledo, 669 - 9º andar, São Paulo, SP 04039-032, Brazil

**Keywords:** Hematopoietic stem cell, HL-60, Leukemic stem cell, Hydrogen peroxide

## Abstract

Over the last few years, studies have suggested that oxidative stress plays a role in the regulation of hematopoietic cell homeostasis. In particular, the effects of hydrogen peroxide (H_2_O_2_) range from hematopoietic cell proliferation to cell death, depending on its concentration in the intracellular milieu. In this work, we evaluated the effects of an oxidative environment on normal and leukemic hematopoietic cells by stimulating normal human (umbilical cord blood) and murine (bone marrow) hematopoietic cells, as well as human myeloid leukemic cells (HL-60 lineage), upon H_2_O_2_ stimulus. Total cell populations and primitive subsets were evaluated for each cell type. H_2_O_2_ stimulus induces HL-60 cell death, whereas the viability of human and murine normal cells was not affected. The effects of H_2_O_2_ stimulus on hematopoietic stem/progenitor cell subsets were examined and the normal primitive cells were found to be unaffected; however, the percentage of leukemic stem cells (LSC) increased in response to H_2_O_2_, while clonogenic ability of these cells to generate myeloid clones was inhibited. In addition, H_2_O_2_ stimulus caused a decrease in the levels of p-AKT in HL-60 cells, which most likely mediates the observed decrease of viability. In summary, we found that at low concentrations, H_2_O_2_ preferentially affects both the LSC subset and total HL-60 cells without damage normal cells.

## Introduction

Hematopoiesis is a continuous process that occurs throughout the life of an organism in which blood cells are produced in an organized and hierarchical process of development initiated in the bone marrow by hematopoietic stem cells (HSC) [1]. Several regulators of hematopoiesis, such as cytokines, G-protein-coupled receptor agonists, vitamins and reactive oxygen species (ROS), contribute to the regulation of hematopoietic cell homeostasis [2-5].

Recent studies have suggested that oxidative stress can regulate hematopoiesis [5]. The participation of ROS in stem cell renewal [6], differentiation [7,8] and proliferation [9,10] has been under investigation. Decomposition of hydrogen peroxide (H_2_O_2_) by catalase increases granulocytes and HSC number in long-term bone marrow cultures [6]. In addition, it was shown the existence of two distinct HSC population based on their ROS content, where HSC ROS^low^ population, but not HSC ROS^high^, exhibits the characteristics of a typical HSC [7]. The importance of oxidative stress regulation has also been described for the differentiation process. In erythropoiesis, ROS generation is important because its accumulation results in hemolysis and shortened red blood cell lifespan [11,12]. These and other reports demonstrate the importance of ROS in the biology of HSC.

Beyond ROS effects on the normal hematopoietic system, similar hematopoietic cells modulation by ROS has been observed in cancer cells, especially in myeloid leukemias [5,13,14]. The best-explored effect of ROS on cancer cells is its capability to harm or kill them, e.g., by the direct toxicity of H_2_O_2_ and nitric oxide [15-17]. Many classes of antineoplastic agents that generates a high level of oxidative stress in biological systems [18], e.g., the synthetic retinoid Fenretinide and diallyl disulfide, promote ROS-dependent cell death in leukemic lineages [19,20].

However, cell death or differentiation ROS-induced is not entirely similar among cell types. A rare primitive cellular population in leukemia, called leukemic stem cells (LSC), contains characteristics of healthy HSC and also exhibits increased resistance to anti-cancer treatments [21,22]. A primitive CD34^+^ subpopulation of a human erythroleukemia multidrug-resistance cell lineage (K562), possesses greater resistance to imatinib than the majority CD34^-^ population sensitive to the elevated ROS levels by the combination of simvastatin and imatinib [23]. However, no studies have compared the direct effects of ROS on normal HSC and LSC.

In this work, it was investigated the behavior of normal and leukemic cells in an oxidative environment by stimulating them with H_2_O_2_. Herein we compared the effects of H_2_O_2_ on the total and primitive subsets of hematopoietic cells from different sources (mouse bone marrow, human cord blood and leukemic HL-60 cells).

## Material and methods

### H_2_O_2_ solution preparation

For stock solutions, H_2_O_2_ (Merck) was serially diluted in phosphate-buffered saline (PBS) to obtain the following concentrations: 10^-1^, 10^-2^ and 10^-3^ M. Before being applied to the cultured cells, H_2_O_2_ solutions were filtered with a 0.22-μM filter (Millipore) and stored at 4°C and protected from light.

### Murine normal hematopoietic cell extraction and culture

The femurs from three-month-old male C57BL/6 mice were extracted, and bone marrow cells were collected in laminar flux by flushing the cells with Iscove’s Modified Dulbecco’s Medium (IMDM) supplemented with 12.5% fetal bovine serum (FBS), 12.5% fetal calf serum (FCS) and 1% antibiotics (penicillin and streptomycin). The cells were centrifuged, counted in a Neubauer chamber and placed into multiwell plates at concentrations of approximately 1×10^6^ cells/ml for the stimulus with H_2_O_2_ at different concentrations, according to the assay. After 8 h of incubation with H_2_O_2_, the cells were collected, washed and separated for analyses. The mice were supplied by the INFAR/UNIFESP Animal Facility. All of the experiments were approved by the Animal Care Ethics Committee of the Federal University of Sao Paulo (1890/09) and were conducted in accordance with the Brazilian Guide for the Care and Use of Laboratory Animals (Federal Law 11794/2008), which is equivalent to the National Institutes of Health guidelines.

### Human normal hematopoietic cell collection and culture

Human umbilical cords from healthy donors were collected after informed patient consent, as approved by the local Ethical Committee of the Federal University of Sao Paulo. Briefly, the placenta was detached after cord clamping and placed in a sterile bowl. A syringe was used to collect the umbilical cord blood (UCB) into a tube containing heparin. Following collection, the cells were manipulated in a sterile environment. The cells were washed in phosphate-buffered saline plus ethylenediaminetetraacetic acid solution (PBS-EDTA) solution 4–6 times to remove as many red cells as possible. To separate the fraction of mononuclear cells, Ficoll-Hypaque (density 1.077 g/ml) centrifugation was used (at 1000 rpm for 30 min) [24]. The mononuclear cells were then counted and placed into multiwell plates at a concentration of approximately 10^6^ cells per well to be stimulated with the appropriate concentration of H_2_O_2_. Twenty-four hours after stimulation, the cells were collected, washed and separated for analyses. Ethical approval number: 0225/10.

### Hematopoietic tumor cell culture

HL-60 cells, from a promyelocytic leukemia immortalized cell lineage, were cultured in RPMI medium supplemented with 10% FBS and antibiotics. When the cells reached approximately 80% confluence, they were plated in multiwell plates at a concentration of 1×10^5^ cells/ml and stimulated with H_2_O_2_. Twenty-four hours after stimuli, the cells were collected, washed and separated for analyses.

### Cellular viability assay

The cells from the three described sources were labeled with annexin-V and 7-amino-actinomycin D (7-AAD) to evaluate the apoptotic rate of the cells after stimulation with increasing concentrations of H_2_O_2_, ranging from 0 μM (control) to 100 μM. Data acquisitions were performed using flow cytometry. All cytometry analyses in this study were performed on a FACSCalibur (Becton Dickinson) flow cytometer using Cell Quest (Becton Dickinson) and FlowJo 7.6.4 (Tree Star) software.

### Granulocyte-macrophage colony-forming unit (CFU-GM) assay

Mouse CFU-GM assays were performed by mixing 2×10^4^ total bone marrow cells in Methocult medium (M3534, Stem Cell Technologies, USA). For both HL-60 and UCB human cells, H4100 Methocult medium (Stem Cell Technologies, USA) was used, and 10 ng/ml of granulocyte colony-stimulating factor was added to each sample containing 2×10^4^ cells. Each mixture was placed in a 35-mm petri dish, according to the manufacturer’s instructions. The cells were cultured at 37°C in a 5% CO_2_ atmosphere under saturated humidity for 7–14 days. At the end of the incubation period, colonies containing more than 50 cells were counted using an inverted microscope at 40x magnification.

### Immunophenotyping

Antibody cocktails were used to sort primitive and mature cells. For mouse stem cells (m-HSC) and progenitor cells (m-HPC), the following antibodies were used: Ter119 (Ly-76), Mac-1 (myM1/70), Gr-1 (RB6-8C5), B220 (RA3-6B2), CD3 (145-2C11) and Flk-2 (A2F10) (Lin-PE), CD90 (Thy1.1)-FITC (HIS51), Sca-1-PE-Cy7 (Ly-6A/E-D7) and c-Kit-APC (2B8). Myeloid mature progeny cells were labeled with Mac-1-PE-Cy7 and Gr-1-PE. The analyzed phenotypes of m-HSC and m-PHC were Lin^-^Flk2^-^CD90^Low^Sca-1^+^c-Kit^+^ and Lin^-^Flk2^-^CD90^Low^Sca-1^-^c-Kit^+^. For UCB and HL-60 cells, the following antibodies were used: CD38 (HIT2), CD33 (WM53), CD2 (RPA-2.10), CD3 (HIT3a), CD7 (eBio124-D1) (Lin-PE), CD34-APC (581), CD15-FITC (MMA) and CD11b-PE-Cy7 (ICRF44). The analyzed primitive phenotypes were CD34^+^ (h-HPC for UCB cells and LPC for HL-60 cells) and CD34^+^Lin^-^ (h-HSC for UCB cells and LSC for HL-60 cells). Mature cells were evaluated based on the expression of the following myelomonocytic markers: CD11b and Gr-1 for BM cells and CD15 and CD11b for UCB and HL-60 cells. The cells were incubated with their respective antibodies for 20 min, then were washed and resuspended with 200 μl of PBS for cytometry data acquisition of 200.000 (primitive populations) or 50.000 (mature progeny) events. For primitive subsets, a gate strategy analysis was used; for the mature subsets, the quadrant was used to divide cells according positively to each channel based on samples of calibration control.

### Protein activation

To measure the basal activation of proteins associated with hematopoiesis, total bone marrow cells (3×10^6^ cells/sample) were obtained from mouse femurs and fixed with 2% paraformaldehyde for 30 min. The cells were washed with 0.1 M glycine in PBS, permeabilized with 0.001% Triton X-100 in PBS for 15 min and washed with PBS. Subsequently, the cells were incubated for 2 h with 4 μg/ml of the appropriate antibodies diluted in PBS containing 1% BSA. Antibodies specific for phosphorylated (p) proteins (activated forms) were used. The following rabbit IgG primary antibodies were used: p-AKT Thr308, p-PLCγ2 Tyr759 and anti-p-ERK1/2 Thr202/Tyr204 (Cell Signaling Technology, USA). After incubation with the primary antibodies for 2 h, the samples were incubated with a goat anti-rabbit IgG Alexa Fluor 488-conjugated secondary antibody (Invitrogen, USA) for 1 h. For primitive identification after protein labeling, antibody cocktails were used as described above.

### Statistical analyses

Fluorescence intensity was quantified using the Geometric Mean (Gm). Student’s *t-*tests were used for comparisons between two groups. To compare data among more than two groups, ANOVA was performed, followed by Bonferroni’s test. Values are presented as the mean ± SEM. Differences were considered significant when *P* < 0.05.

## Results

### HL-60 cell viability is specifically targeted by H_2_O_2_

**Figure 1:**
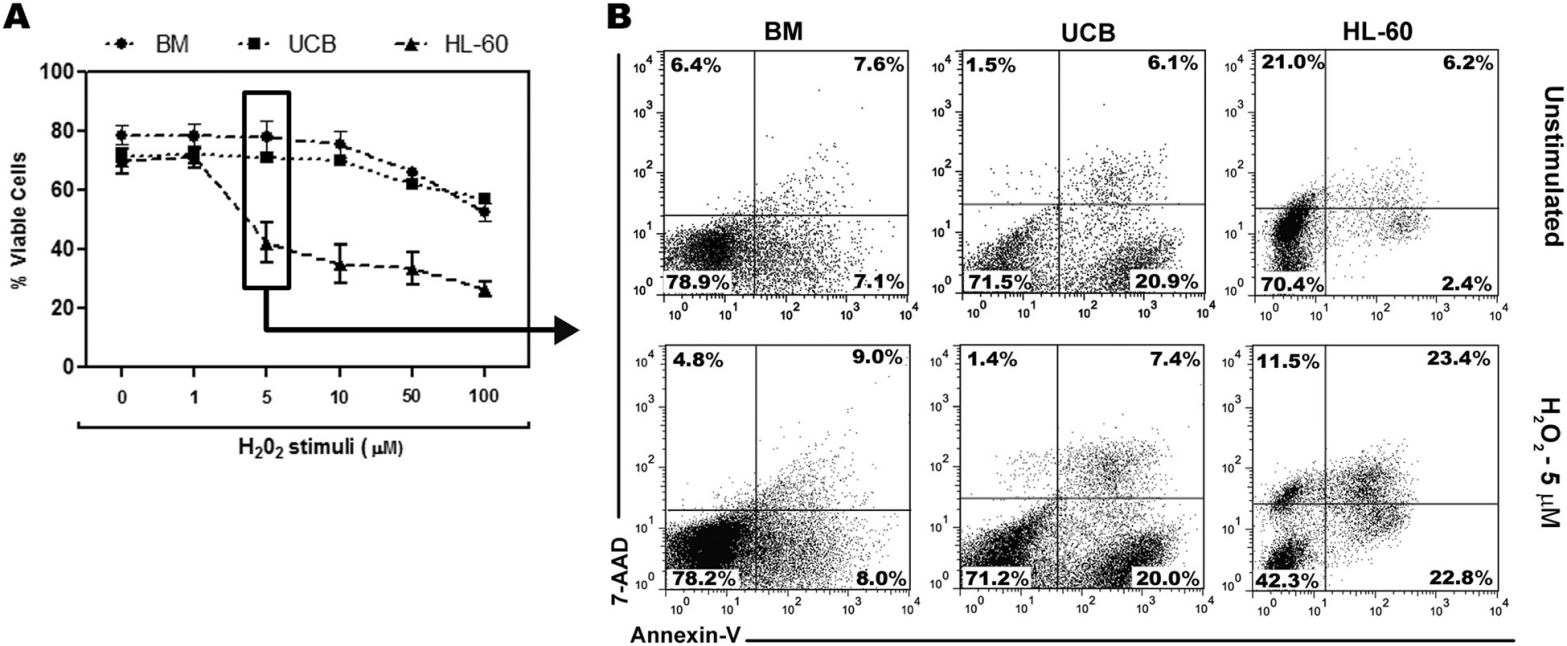
**The HL-60 leukemic cell lineage is more sensitive to H**_**2**_**O**_**2 **_**than normal hematopoietic cells.** After stimuli with H_2_O_2 _*in vitro (*ranging from 0 to 100 μM), BM, UCB and HL-60 cells were collected and labeled with annexin-V and 7-AAD for the quantification of viable and dead cells by flow cytometry. **(A)** The dose–response curves show that HL-60 cells display increased sensitivity to H_2_O_2_. **(B)** Representative dot plots showing the percentage of dead cells for each cell type. The data are expressed as the mean ± SEM of independent experiments performed in duplicate. BM n = 5, UCB and HL-60, n = 3 (3 independent experiments in duplicate).

Initially, the cytotoxicity of H_2_O_2_ in different cell types (human mononuclear cells from UCB, HL-60 cells and BM from mice) was tested, and dose–response curves were built. The cells were stimulated with increasing concentrations of an exogenous source of H_2_O_2_ for 24 h (UCB and HL-60) or 8 h (BM). Interestingly, even at the lowest concentration of H_2_O_2_ (5 μM), the HL-60 cells were affected, whereas normal populations from UCB and BM cells were not affected (Figure 1A). Above 5 μM, all cell types showed a progressive decrease in viability (Figure 1A). Cellular stimulus with 5 μM of H_2_O_2_ induces apoptosis of HL-60 cells, and also resulted in the increase of annexin^+^7AAD^+^ cells, that can be related to both apoptosis and necrosis. Higher concentrations, such as 100 μM, were particularly effective at killing HL-60 cells (approximately 60% less viability than unstimulated HL-60 cells) compared with normal UCB and BM cells.

### H_2_O_2_ triggers different effects in normal and leukemic stem cell populations

**Figure 2:**
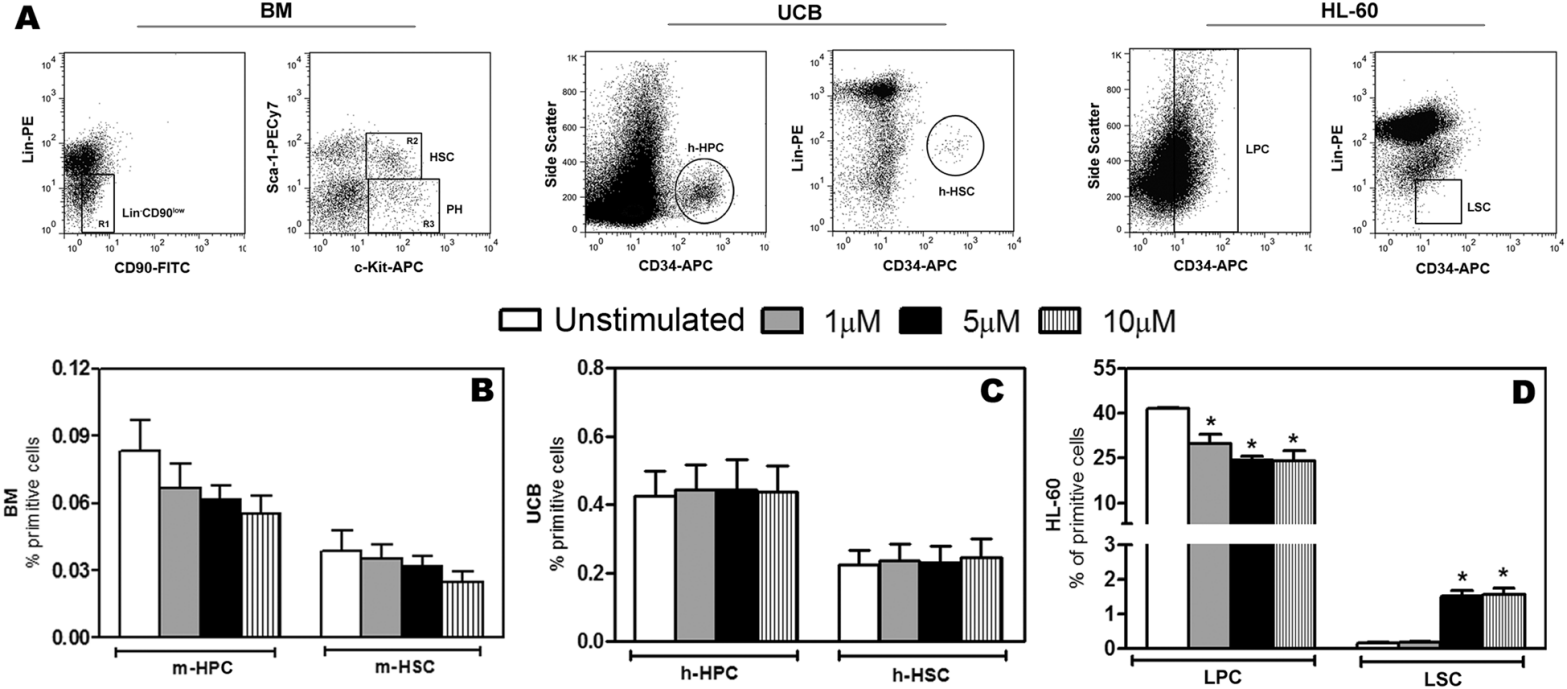
**Primitive cells from HL-60 human myeloid leukemic cells are affected by H**_**2**_**O**_**2**_**.** BM, UCB and HL-60 cells stimulated 1, 5 and 10 μM of H_2_O_2 _*in vitro* were labeled with specific mAbs for identification of the primitive subsets. **(A)** Representative dot plots from the gate strategy analysis used to select the populations of interest: m-HPC (Lin^-^Flk2^-^CD90^Low^Sca-1^-^c-Kit^+^), m-HSC (Lin^-^Flk2^-^CD90^Low^Sca-1^+^c-Kit^+^), h-HPC and LPC (CD34+), h-HSC and LSC (CD34^+^Lin^-^). **(B and C)** H_2_O_2_ did not alter the percentages of both murine and human hematopoietic progenitors and HSC **(D)** H_2_O_2_ reduces the LPC population and increases the LSC population. The data are expressed as the mean ± SEM of independent experiments performed in duplicate. BM n = 5, UCB and HL-60 n = 3 (3 independent experiments in duplicate). *P < 0.05, ANOVA.

Because cell fate decisions can be modulated by several cellular intrinsic and extrinsic factors, possible alterations in the subset of HSC from BM, UCB and HL-60 cells lineage (Figure 2A) were evaluated after stimulus with H_2_O_2_. The percentage of HSC and progenitors cells from BM and human HSC from UCB were unaffected by H_2_O_2_ stimulus (Figure 2B and C). The total CD34^+^ population of the HL-60 cells (LPC) was diminished at all concentrations tested; however, 5 and 10 μM of H_2_O_2_ induced an increase in the LSC population (Figure 2D).

In addition, the ability of primitive cells to form clones was evaluated after stimulus with 5 μM of H_2_O_2_ once at this concentration it was observed a preferentially effect on HL-60 viability, as well as its impact on LSC subset. H_2_O_2_ promoted an increase of BM myeloid clones, a reduction of approximately 70% in the number of colonies formed by UCB cells and a total inhibition of HL-60 cell colony formation (Table 1).

**Table 1:** **H**_**2**_**O**_**2 **_**leukemic primitive cell clonogenic capacity**

	**Unstimulated**	**5 μM H**_**2**_**O**_**2**_
**BM**	68 ± 2	86 ± 7*
**UCB**	45 ± 4	12 ± 2*
**HL-60**	310 ± 4	0*

A methylcellulose clonal assay was used to quantify clonogenic capacity of primitive cells obtained from BM, UCB and HL-60 lineage. Clonogenic capacity of BM was increased, showing that H_2_O_2_ induced the improvement of the capability of these cells to generate their progeny. UCB colonies decreased around 70% while HL-60 stimulated with H_2_O_2_ was not able to generate clones. The data are expressed as the mean ± SEM of 4 experiments. *P< 0.05, ANOVA.

### Differentiation is not affected by H_2_O_2_ at low concentrations

**Figure 3:**
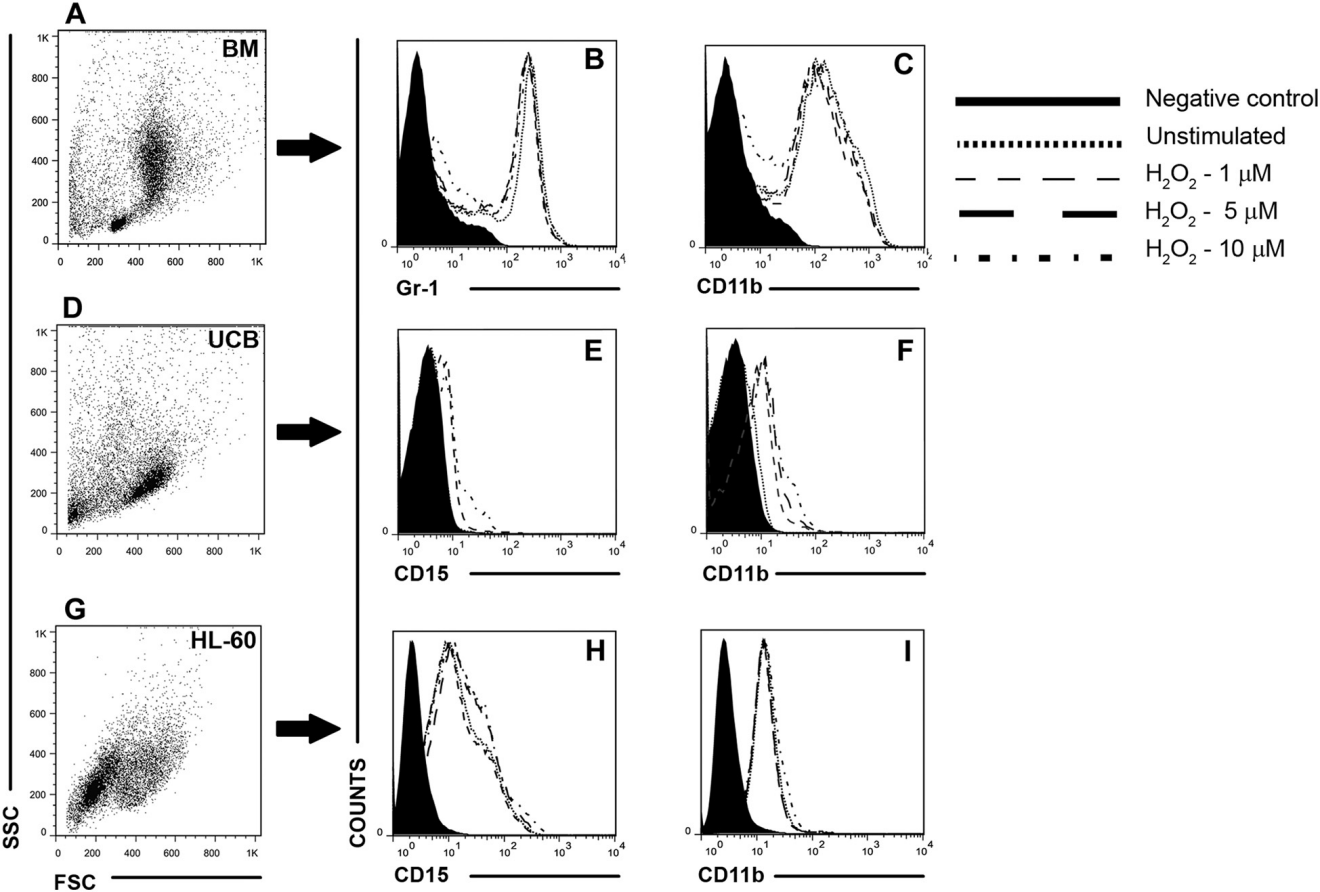
**H**_**2**_**O**_**2 **_**did not alter the expression of myelomonocytic markers. (A, D, G)** Representative dot plots of evaluated cells (forward scatter vs. side scatter) of BM, UCB and HL-60 cells, respectively. **(B and C)** Histograms demonstrating the overlap of CD11b and Gr-1 expression in entire BM cell population, and also the higher constitutive expression of these markers in comparison to UCB and HL-60 cells. **(E, F, H and I)** Histograms demonstrating the overlap of CD15 and CD11b expression in UCB and HL-60 cells, respectively. Any change in the expression of mature markers among unstimulated and H_2_O_2_ stimulated groups was observed. All data are representative responses of at least 3 experiments performed in duplicate.

To verify whether there was a correlation between the observed alterations in primitive cellular subsets with the induction of differentiation of the cells by H_2_O_2_, the expression of mature myeloid markers was analyzed. The constitutive expression of the myelocytic markers differed among the cell types that were evaluated. Mouse bone marrow cells showed high expression of Gr-1 and CD11b (Figure 3B-C), while CD11b expression in UCB cells was lower than CD15 expression (Figure 3E-F), and both markers were little expressed in HL-60 cells (Figure 3H-I). Nevertheless, H_2_O_2_ did not induce differentiation of any of the hematopoietic cell types evaluated (Figure 3B-C-E-F-H-I).

### AKT phosphorylation is decreased by H_2_O_2_ in HL-60 cells

**Figure 4:**
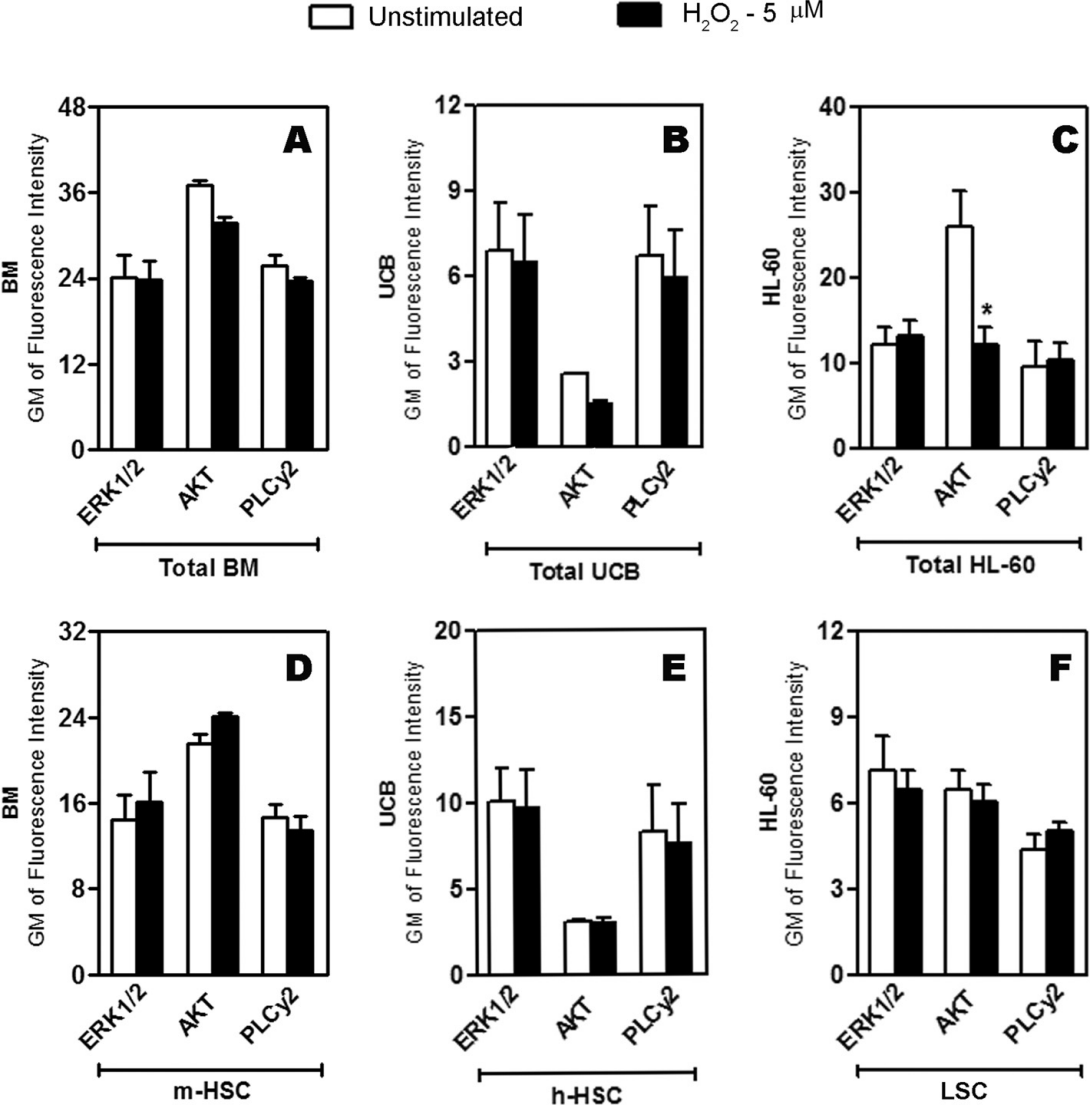
**AKT phosphorylation is decreased by H**_**2**_**O**_**2 **_**stimulus in total HL-60 cells.** The cells were stimulated with 5 μM of H_2_O_2_, fixed, permeabilized and labeled with anti-phospho proteins to verify the activated status of ERK1/2, AKT and PLCγ2 by flow cytometry **(A-C)** BM, UCB and HL-60 cells and in the **(D-F)** stem cell subsets of each population. The fluorescence intensity of p-ERK1/2, p-AKT and PLCγ2 measured showed a significant decrease of p-AKT in total HL-60 cells. The data are presented as Gm. The data are expressed as the mean ± SEM of 3 independent experiments performed in duplicate. *P < 0.05, ANOVA.

ERK, AKT and PLCγ2 are known to be involved in the signaling cascades that control survival, growth and differentiation of cells during both normal and tumoral hematopoiesis [25-27]. Therefore, we investigated whether these proteins mediated the effects caused by H_2_O_2_ in total cells and in the primitive cells subset. As shown in Figure 4, neither ERK1/2 nor PLCγ2 activity was altered by H_2_O_2_ stimulus in any group. However, AKT phosphorylation was decreased after H_2_O_2_ stimulus in total HL-60 cells (Figure 4C), whereas AKT phosphorylation in the LSC subset was not affected (Figure 4F).

## Discussion

H_2_O_2_ is one of the most versatile oxidants. Because of its high cell permeability, it can act as an intracellular second messenger molecule [28]. At the micromolar range, H_2_O_2_ can induce alterations in the phosphorylation of specific regulatory proteins, leading to activation of signaling pathways and transcription factors, as well as genes involved in antioxidant defenses, under stressful conditions, it can promote cell death [29].

Hematopoietic cells have been suggested to be particularly vulnerable to ROS, which can trigger malignancies, such as lymphomas and sarcomas, in hematopoietic tissues [5]. However, ROS can also serve as important molecules that control stem cell fate [30]. The improvement of HSC and progenitor cell expansion is achieved when H_2_O_2_ is scavenged by catalase [6] or N-acetyl-cysteine [31]; however, when H_2_O_2_ is present, cell adhesion to the niche is suppressed and HSC detach from the osteoblastic niche, thereby inhibiting the quiescent state [32]. By contrast, during the reconstitution of hematopoiesis after lethal irradiation, the regulation of vascular cell adhesion molecule-1 expression on endothelial cells, which is related to the proliferation of stem/progenitor cells, is a ROS-dependent process [33].

Although the role of ROS in the biology of normal hematopoietic cells is not completely understood, its role in the therapeutic field of hematopoietic cancer cells has been better addressed. It is known that anti-cancer drugs often augment ROS formation, leading the cancer cells to undergo apoptosis; however, these drugs also cause bone marrow cytotoxicity, which is an off-target effect that may be related to the inability of drugs to differentiate between malignant and normal cell populations, a significant barrier to treatment efficacy [34]. In the present work, apoptosis and necrosis was induced by H_2_O_2_ at low concentrations (5 μM) in human HL-60 leukemic cells, while the viability of normal cells from both murine bone marrow and human UCB was not significantly affected (Figure 1). Previous studies have shown that 75 μM of H_2_O_2_ induces necrosis of in lymphoma cell lineages by depleting cellular ATP stores, while at lower concentrations, H_2_O_2_ induces apoptosis [35]. It is likely that the significant effects of H_2_O_2_ on cancer cells at the micromolar range are due to its constitutively higher intracellular ROS content than normal cells [36,37]. At nanomolar concentrations, H_2_O_2_ does not have significant effects on the cellular viability of HL-60 cells (data not shown). In mouse total bone marrow cells, a significant amount of cell death was observed when the cells were stimulated with 500 μM of H_2_O_2_, with more than 75% non-viable cells (data not shown).

Beyond the analysis of the effects of H_2_O_2_ on cell survival, we also examined its potential effects on cellular differentiation. The increase in ROS in normal HSC is related to the increase of cell cycle turnover and death and commitment to the myeloid lineage [9]. As shown in Figures 2 and 3, H_2_O_2_ induced a decrease in the m-HPC and m-HSC populations, but these differences were not significant. However, correlating these data with the increased number of colonies formed upon 5 μM of H_2_O_2_, it is possible that the improvement of BM clonogenic capacity has been occurred in a cellular myeloid commitment response, considering the progressive decrease of m-HSC and the induced increase in granulocytes/macrophages cell colonies (Table 1), which was not observed in terms of terminal differentiation (Figure 3B-C) probably due to the short period of evaluation. On the other hand, UCB capacity of forming colonies was decreased (Table 1) even though the percentage of h-HPC and h-HSC remained unaltered. In contrast, the percentages of LPC diminished in response to all concentrations tested, while the percentage of the most primitive HL-60 cells (LSC) increased significantly after stimulus with 5 and 10 μM (Figure 2D), the same concentrations that caused the most HL-60 cell death. Despite the increase in the percentage of LSC, the clonogenic survival of HL-60 cells was greatly affected because any GM-CFU were formed by the H_2_O_2_-stimulated cells (Table 1), showing that although this cell population has increased, they were not functional once they were not able to generate their progeny, resulting in its accumulation. Our data shows that the manner in which the cancer stem cells respond to redox alterations is distinct from the more committed subset of cells in the HL-60 lineage. It is plausible to consider the existence of a refined defense mechanism in these cells that confers a protective advantage. These findings are quite interesting, once the distinct behavior of normal and leukemic cells upon stimulus with low concentration H_2_O_2_ is put into perspective, and such a feature can be exploited in an unhealthy physiologic system, in which both cellular types are present at the same microenvironment.

To address the mechanism by which H_2_O_2_ exerts its effects in normal and leukemic cells, the activated (i.e., phosphorylated) state of known signaling mediators of proliferation, differentiation and survival in normal and tumoral hematopoiesis [25] was determined. Despite the importance of PLCγ2 and ERK1/2 in the hematopoiesis process [26,27], any change in the phosphorylation of these proteins was observed after stimulus with H_2_O_2_. However, a significant alteration was found in the phosphorylation of the protein AKT, which was inhibited in the total HL-60 population (Figure 4C) but not in LSC (Figure 4E). This result is in agreement with previous studies showing that AKT is necessary for the survival of both normal [38] and tumor cells [39] from hematopoietic system. In addition, it is possible that that together with the maintenance of p-AKT level in LSC, other signaling proteins are playing a prosurvival role in these cells, e.g. the participation of p38MAPK which has been shown to be involved in the regulation of cell checkpoints for the promotion of cancer cells survival [40,41].

The different behavior of normal cells and leukemic cells in response to ROS stimuli observed in this work can be explained by an important aspect that differs between them: the constitutively higher ROS level in cancer cells compared with normal cells. A clinical trial meta-analysis based on the administration of exogenous antioxidants to patients resulted in increased mortality, which was most likely caused by an inhibition of cancer cell apoptosis [36] and also non-significant benefits of the antioxidant response [42]. Therefore, it is unclear how advantageous an improvement in antioxidant defenses as adjuvant will be in the clinical setting. On the contrary, ROS modulation-based therapy is an alternative intervention approach to antioxidant therapy. This approach is expected to elevate chemical damage in cells with preexisting oxidative imbalance (cancer cells) without affecting the adjacent normal cells [43]. Furthermore, it has been suggested that the reason that many current chemotherapeutic agents fail is their inability to selectively target the cancer stem cells [44]; thus, the best strategy would affect only the cancer cell population subset.

In summary, we found that H_2_O_2_ preferentially affects leukemic cells by decreasing their viability, quiescent state and clonogenic capability via the modulation of survival signaling through decreased AKT activity. From a clinical point of view, treatments based on the ROS modulation levels are a promising therapeutic tool, once the redox state-induced alterations can cause selective hematopoietic cell fate, sensitizing leukemic cells to death. Further studies are required to further understand the role of ROS in the modulation of hematopoiesis, thereby enabling the development of redox-based therapeutic interventions in favor of patients with myelodysplastic syndromes.

## Competing interests

The authors declare that they have no competing interests.

## Authors’ contributions

ANP participate in all stages of the assays development and preparation of manuscript. TAMC carried out mostly HL-60 cell lineage assays. CCD helped in cells immunolabeling. CSTO and LPME were responsible for the collection of human cord blood cells. EJPG and ATF scientifically conducted the study and corrected the manuscript. All authors read and approved the final manuscript.
